# Decoding lifespan secrets: the role of the gonad in *Caenorhabditis elegans* aging

**DOI:** 10.3389/fragi.2024.1380016

**Published:** 2024-03-26

**Authors:** Andre Pires da Silva, Rhianne Kelleher, Luke Reynoldson

**Affiliations:** School of Life Sciences, University of Warwick, Coventry, United Kingdom

**Keywords:** diapause, evolution of aging, lipids, germline, reproduction

## Abstract

The gonad has become a central organ for understanding aging in *C. elegans*, as removing the proliferating stem cells in the germline results in significant lifespan extension. Similarly, when starvation in late larval stages leads to the quiescence of germline stem cells the adult nematode enters reproductive diapause, associated with an extended lifespan. This review summarizes recent advancements in identifying the mechanisms behind gonad-mediated lifespan extension, including comparisons with other nematodes and the role of lipid signaling and transcriptional changes. Given that the gonad also mediates lifespan regulation in other invertebrates and vertebrates, elucidating the underlying mechanisms may help to gain new insights into the mechanisms and evolution of aging.

## Introduction

Aging, characterized by a time-dependent deterioration of physiological function, is a phenomenon that is almost universally observed in biology (Jones et al., 2014). Pioneering work using the nematode *C. elegans* has provided insights into the genetics of aging (Klass, 1977; Johnson and Wood, 1982; Klass, 1983; Friedman and Johnson, 1988). These studies showed that single gene mutations can greatly extend *C. elegans* lifespan, sometimes up to tenfold compared to its normal lifespan (Friedman and Johnson, 1988; Kenyon et al., 1993; Ayyadevara et al., 2008). Several pathways, including the highly conserved insulin signaling pathway and a germline signaling pathway (Kenyon, 2011; Ghazi, 2013), are involved in modulating aging (Soo et al., 2023).

Compared to the insulin signaling pathway, the germline signaling pathway is relatively understudied (Lemieux and Ashrafi, 2016). The initial discovery was based on removing germline precursor cells in *C. elegans* larvae (Hsin and Kenyon, 1999). These laser-ablated nematodes, which have an intact somatic gonad without germline cells, reach adulthood and live substantially longer than non-ablated animals (Hsin and Kenyon, 1999). These findings initially supported a theory of aging stating that energy resources could be diverted from reproduction to somatic maintenance to extend lifespan [reviewed in (Kirkwood, 1991; Lemaitre et al., 2015)]. However, the complete removal of the reproductive system (both the somatic gonad and the germline) does not extend lifespan, contradicting this “resource allocating” theory of aging (Hsin and Kenyon, 1999). These ablation experiments suggest that whereas somatic gonad signals may lengthen lifespan, they are counteracted by lifespan-shortening germline signals (Hsin and Kenyon, 1999).

Mutants that genetically mimic laser-ablated animals have been used to study the regulation of lifespan extension in germline-less animals. The use of such mutants allows the generation of a large number of animals lacking a germline. Biochemical analysis of germlineless mutants indicates they are rich in triglyceride/phospholipid content (O’Rourke et al., 2009), suggesting that the lifespan extension of germlineless worms may involve changes to fat metabolism.

Naturally, in the wild, there are no animals lacking germline. Therefore, it is crucial to determine if the conditions that prevent germline proliferation (e.g., starvation) and lead to extended lifespan involve the same regulatory pathways as those observed in lab-engineered germline-less animals. These studies will give insights into aging mechanisms and theories of aging alike. This review aims to address the mechanisms behind the increased lifespan of mutants lacking proliferating germ cells, connecting these findings with recent theories of aging, identifying gaps in the literature, and suggesting potential future research directions.

## Senescent pathologies in aging *C. elegans*

*C. elegans* is usually maintained in genetically homogenous populations of self-fertilizing hermaphrodites. They propagate on agar plates, using the bacterium *Escherichia coli* as a food source (Stiernagle, 2006). In these conditions, the hermaphrodite lives for an average of 18 days at 20°C. In its natural habitat on decaying vegetable matter, *C. elegans* feeds on uncharacterized bacterial and unicellular eukaryotes (Félix and Duveau, 2012; Frézal and Félix, 2015). Although *C. elegans* lifespan has been determined in complex environments (Van Voorhies et al., 2005), its lifespan in the original habitat and native food is not known. Additionally, it is unclear if *C. elegans* displays signs of senescence in its natural environment (Nussey et al., 2013).

In the laboratory, the aging *C. elegans* hermaphrodite displays multiple pathologies, including the degeneration of the germline, pharynx, body wall muscle, vulva and intestine (Garigan et al., 2002; Herndon et al., 2002; McGee et al., 2011; de la Guardia et al., 2016; Leiser et al., 2016), ectopic deposition of lipids (Palikaras et al., 2017; Palikaras et al., 2023) and yolk (lipoproteins) (Ezcurra et al., 2018; Kern et al., 2023; Spanoudakis and Tavernarakis, 2023). These pathologies start relatively early, with some already apparent on only the third day of adulthood (Herndon et al., 2002; Ezcurra et al., 2018). Additionally, measures of health, such as vigor of movement, effective pharyngeal pumping, and resistance to stressors (including oxidative stress or thermotolerance), decline with age in *C. elegans* (Bansal et al., 2015). It must be noted, however, that measures of health in *C. elegans* have not yet been strictly defined (Bansal et al., 2015).

*C. elegans* males tend to live longer than hermaphrodites, provided they are kept in isolation (McCulloch and Gems, 2003; Ancell and Pires-daSilva, 2017). Because male proportions are low in the laboratory and nature (Frézal and Félix, 2015), combined with their tendency to kill each other when raised in groups, and their propensity to escape the plates, determination of their lifespan is often excluded (Gems and Riddle, 2000). In a few studies designed to characterize the pathological changes in aging males, neither intestine (Ezcurra et al., 2018) nor germline disintegration occurs (de la Guardia et al., 2016), and motor decline is detected before any visible morphological changes (Guo et al., 2012).

## Comparing germline-ablated animals and germline-less mutants

The first larval stage of *C. elegans* contains two germline precursor cells, named Z2 and Z3 (Kimble and Hirsh, 1979). Removal of these cells by laser cell ablation results in an adult with an intact somatic gonad lacking oocytes and sperm. *C. elegans* hermaphrodites lacking a proliferating germline are long-lived (Hsin and Kenyon, 1999) and resistant to stress (Arantes-Oliveira et al., 2002; Sinha and Rae, 2014). In *C. elegans* males, the ablation of germline precursor cells results in a slight life extension when grown on agar plates (Arantes-Oliveira et al., 2002), but not when kept in liquid culture (McCulloch, 2003).

In the wild-type *C. elegans* adult, the proliferation of the germline stem cells is mediated by signals from the distal tip cells of the somatic gonad [for review, see (Hubbard and Schedl, 2019)]. Removing these somatic cells causes premature differentiation of the germline stem cells into gametes (Kimble and White, 1981). *glp-1*, a member of the Notch receptor family (Yochem et al., 1988; Austin and Kimble, 1989; Kimble and Simpson, 1997) expressed in the germline (Cinquin et al., 2015; Gutnik et al., 2018; Sorensen et al., 2020), is required to keep stem cells in an undifferentiated state. Thus, *glp-1* loss-of-function mutants mimic the Z2/Z3-ablated animals because both lack proliferating and undifferentiated germ cells. The most commonly used mutants are temperature-sensitive and are subjected to the restrictive temperature during larval stages to induce their phenotype (Arantes-Oliveira et al., 2002). Similar to the Z2/Z3-ablated animals, *glp-1* mutant hermaphrodites are long-lived (Hsin and Kenyon, 1999), display delayed senescent phenotypes (Palikaras et al., 2017) and are stress-resistant (Arantes-Oliveira et al., 2002; Miyata et al., 2008; Alper et al., 2010; Soo et al., 2023).

Additional examples of long-lived mutants with no proliferating germ cells include *glp-4*, *mes-1,* and *pgl-1* (Beanan and Strome, 1992; Arantes-Oliveira et al., 2002; Tatar, 2002; Curran et al., 2009). There are only a few studies with the *pgl-1* mutant; therefore, we will not discuss them further. *glp-4* codes for a tRNA synthetase (Rastogi et al., 2015). Similar to *glp-1* mutant animals, mutants for a loss-of-function temperature-sensitive allele of the gene *glp-4* (allele *bn2*) share many phenotypes: the germline does not proliferate (Beanan and Strome, 1992), fat storage is altered (Wang et al., 2008) and are resistant to stress (Alper et al., 2010; Greer et al., 2010; TeKippe and Aballay, 2010; Labbadia and Morimoto, 2015). *glp-4* (*bn2)* animals show delays to pathological signs of senescence (Ezcurra et al., 2018; Kern et al., 2023) and have an extended lifespan (Arantes-Oliveira et al., 2002; Okuyama et al., 2010; TeKippe and Aballay, 2010), although there are reports that contradict this finding (Tohyama et al., 2008; Greer et al., 2010). For instance, *glp-4* animals have a wild-type lifespan when grown on live bacteria but show an extended lifespan only when grown on dead *E. coli* (TeKippe and Aballay, 2010).

When raised at the restrictive temperature, *C. elegans* mutants with the temperature-sensitive alleles of *mes-1* do not develop the germline precursors Z2 and Z3 and therefore do not contain germline cells (Strome et al., 1995). Lifespan extension and stress resistance were reported for both hermaphrodites (Arantes-Oliveira et al., 2002; Alper et al., 2010; Wu et al., 2015) and males, although only slightly for the latter (McCulloch, 2003).

The use of genetic mutations to replicate germline ablations has significantly advanced our understanding of the metabolic and genetic changes in animals lacking a proliferating germline (Pu et al., 2017; Wan et al., 2017; Burkhardt et al., 2023; Chaturbedi and Lee, 2023). However, the strengths of using *glp-1* temperature-sensitive alleles, such as *glp-1* (*q224ts*) and *glp-1* (*bn18*) (Austin and Kimble, 1987; Kodoyianni et al., 1992) may affect the interpretation of some studies as they show phenotypes in other tissues that could influence lifespan (Apfeld and Kenyon, 1999; Singh et al., 2011; Entchev et al., 2015; Zhang et al., 2018; Uno et al., 2021). Furthermore, the *glp-4* (*bn2*) mutant has a partial loss of function in the soma (Rastogi et al., 2015). Given that *glp-4* (*bn2*) does not show the same extent of lifespan extension as *glp-1* (TeKippe and Aballay, 2010), it would be beneficial to also include alternative models such as *mes-1* and *pgl-1*, or engineer new strains that allow spatiotemporal control of gene expression of genes that affect the proliferation of germline cells [e.g., (Zhang et al., 2015)].

## Changes in transcriptional control mechanisms following germline removal

The germline removal in *C. elegans* results in the differential expression of thousands of transcripts (Sinha and Rae, 2014; Blackwell et al., 2015) and hundreds of proteins (Krijgsveld et al., 2003; Bantscheff et al., 2004; Pu et al., 2017). Among these are transcriptional regulators previously implicated in modulating lifespan, such as the pro-longevity transcription factors DAF-16 (mammalian FOXO) and DAF-12. The activity of DAF-16 is essential for the increased lifespan of animals with Z2/Z3 ablation (Hsin and Kenyon, 1999). The translocation of DAF-16 from the cytoplasm to the nucleus, a requirement for its function (Lin et al., 2001), relies on the activity of DAF-12. Interestingly, this specific activity of DAF-12 in regulating DAF-16 nuclear localization occurs only when the germline cells are removed (Berman and Kenyon, 2006). Similarly, the kinase MBK-1, the transcription elongation factor TCER-1, and the cytoskeleton adaptor protein KRI-1 modulate DAF-16 activity only in *glp-1* mutants, but not in long-lived mutants of the insulin pathway (Berman and Kenyon, 2006; Mack et al., 2017; Amrit et al., 2019).

DAF-12 is a nuclear hormone receptor similar to the vitamin D receptors found in vertebrates (Antebi et al., 2000). Its activation is mediated by the ligand dafachronic acid (DA), a cholesterol-derived hormone (Motola et al., 2006). However, significant lifespan extension can be induced in animals lacking germline and somatic reproductive tissues by supplementation with DA (Yamawaki et al., 2010). This suggests that the somatic gonad triggers the production of the DAF-12 ligand in animals lacking only the germline (Gerisch et al., 2007). In addition to regulating DAF-16 cellular localization, DAF-12 also activates the fatty acid reductase *fard-1*, a gene required for lifespan extension in animals lacking germline (McCormick et al., 2012).

The intestine is a key site where DAF-16 exerts its effects. While DAF-16 is present in both muscles and neurons, its activity in extending lifespan upon germline removal is specifically required in the intestine (Libina et al., 2003). Targets of DAF-16 include genes involved in proteolysis *rpn-6*, a subunit of the proteasome (Vilchez et al., 2012). DAF-16 can form a complex with the transcription factor HLH-30 (mammalian TFEB), leading to the joint regulation of a shared group of promoters (Lin et al., 2018), or independently regulating their specific targets (Lin et al., 2018). Proteostasis is also regulated by endogenous siRNAs that activate stress-responsive genes through the heat-shock transcription factor HSF-1 (Cohen-Berkman et al., 2020).

Together with TCER, DAF-16 regulates lipid homeostasis (Ghazi et al., 2009; Amrit et al., 2016). Among the genes regulated by these factors are lipases *lipl-1* and *lipl-2* [90, lips-17 {McCormick, 2012 #10478], the fatty acid desaturase *fat-5* (Goudeau et al., 2011; McCormick et al., 2012), and the fatty acid elongase *elo-2* (McCormick et al., 2012). A DAF-16 target, the lipase LIPL-4 (Wang et al., 2008; Mony et al., 2021), activates the nuclear hormone receptor NHR-49 (mammalian PPARɑ) (Folick et al., 2015). NHR-49 is necessary for lifespan extension in *C. elegans* lacking germline, and it upregulates the expression of genes involved in *de novo* fat synthesis (Ratnappan et al., 2014). LIPL-4 also induces autophagy by upregulating the activity of the transcription factor PHA-4 (Lapierre et al., 2011).

The nuclear hormone receptor, NHR-80 (Goudeau et al., 2011), together with NHR-49, is activated by LIPL-4 (Folick et al., 2015). Following a common theme from the transcriptional regulators mentioned above, NHR-80 regulates lipid metabolism by controlling the expression of desaturases, requiring DAF-12 (Goudeau et al., 2011). Likewise, the transcription factor SKN-1 is activated in the intestine upon germline removal and regulates lipid metabolism and stress resistance (Steinbaugh et al., 2015). The activation of SKN-1 is mediated by the generation of redox species and H_2_S, enabled by KRI-1 (Wei and Kenyon, 2016). How exactly KRI-1 changes the redox chemistry is not known.

In summary, fat-processing enzymes are overrepresented in *C. elegans* without a proliferating germline (Wang et al., 2008; Goudeau et al., 2011; McCormick and Kennedy, 2012). Some of those enzymes (e.g., LIPL-4), when constitutively expressed, result in lifespan extension (Wang et al., 2008). Although initially it was proposed that the main benefit of lipids was the result of catabolism processes (Wang et al., 2008), it was later found that the synthesis of lipids was also important (see next section).

## Changes in lipid metabolism in *C. elegans* lacking a proliferating germline

One of the hallmarks of *C. elegans* lacking a proliferating germline is the remodeling of lipid distribution and metabolism (O’Rourke et al., 2009; Wang et al., 2008; Hansen et al., 2013; Bustos and Partridge, 2017; Wan et al., 2019). Lipids are structurally diverse, but share common biophysical properties such as hydrophobicity. They have multiple functions, including roles as components of cellular structures, signaling molecules, and energy storage (Mutlu et al., 2021). *C. elegans* lipid constitution and metabolism were reviewed recently (Watts and Ristow, 2017; An et al., 2023), as well as their role in aging (Papsdorf and Brunet, 2019; Parkhitko et al., 2020; Mutlu et al., 2021; Bresgen et al., 2023).

The cholesterol-derived hormone dafachronic acid (DA) is critical for *glp-1* lifespan extension by enhancing the activity of the transcription factor DAF-12. The enzyme DAF-9, essential for the synthesis of DA, is expressed in the somatic gonad. This evidence is suggestive of the role of DA as the somatic pro-longevity signal in germline-less *C. elegans* (Yamawaki et al., 2010). A simple model is that somatic gonad can stimulate DA production when the germline cells are removed. However, although an initial report indicated an increase in the concentration of DA in *glp-1* mutants (Shen et al., 2012), more sensitive detection methods have disputed these findings (Li et al., 2015). It is thus yet unknown how DAF-12 activity towards the ligand is increased in *glp-1* mutants.

The composition of lipids is influenced by enzymes involved in the processes of fatty acid elongation, desaturation, β-oxidation, and lipase activity. In *glp-1* mutants, the elongase ELO-3 is critical for the activation of SKN-1 (but not for the activation of DAF-16 or HSF-1) (Wang et al., 2021). Synthesis of a lipid intermediate by ELO-3 results in changes in the membrane of lysosomes, ultimately suppressing a nutrient-sensing pathway that promotes the activation of SKN-1 (Wang et al., 2021). Together with NHR-49 (Ratnappan et al., 2014), SKN-1 upregulates genes involved in mitochondrial ß-oxidation (Steinbaugh et al., 2015) in *glp-1* mutants, generating energy and reducing lipid storage. The lysosomal lipase LIPL-4 also increases levels of mitochondrial ß-oxidation, apparently independently of SKN-1. LIPL-4, which is required for lifespan extension in *glp-1* animals (Wang et al., 2008), generates oleoylethanolamide (OEA) (Folick et al., 2015). OEA is a monounsaturated fatty acid that binds to the lipid chaperone LBP-8, which induces nuclear translocation of NHR-80 and NHR-49 (Folick et al., 2015). These transcription factors activate genes in the mitochondria responsible for ß-oxidation (Ramachandran et al., 2019). Consistent with the importance of mitochondrial ß-oxidation for lifespan extension, inhibition of this process in *glp-1* mutants results in a shorter lifespan (Macedo et al., 2020).

The *C. elegans fat-5, fat-6,* and *fat-7* genes encode Δ9-desaturases, which preferentially convert saturated C16:0 and C18:0 fatty acids to the monounsaturated C16:1 and C18:1 fatty acids (Watts and Browse, 2000), have repeatedly been found to be upregulated after removing the germline (Goudeau et al., 2011; Ratnappan et al., 2014; Steinbaugh et al., 2015; Amrit et al., 2016). Dietary supplementation with monounsaturated fatty acids (MUFAs), such as oleic, palmitoleic, or cis‐vaccenic acids, is sufficient to increase lifespan (Han et al., 2017; Lee et al., 2019), and their presence is abundant in other long-lived *C. elegans* mutants (Shmookler Reis et al., 2011). It is not yet clear how MUFAs regulate lifespan, but they have suggested roles in promoting membrane fluidity, enhancing energy storage, and minimizing oxidative stress (Koyiloth and Gummadi, 2022).

The role of lipids in lifespan extension is an area of active investigation, which is complicated by the fact that these molecules are pleiotropic, as well as being very diverse in structure and function. Lipid remodeling also occurs in other sterility mutants (Chaturbedi and Lee, 2023), although it does not result in lifespan extension at 20°C (Kenyon et al., 1993; Arantes-Oliveira et al., 2002; Chaturbedi and Lee, 2023). Recent lipidomic and transcriptomic analysis showed that lower sphingosine levels correlate with a longer lifespan (Chaturbedi and Lee, 2023), but the significance of this correlation still needs to be determined.

## Prolonged lifespan and reproductive quiescence in starved *C. elegans*

Our discussions have so far centered on lifespan extension through germline removal by artificial means. It is interesting to note that lifespan can also extend naturally, particularly under conditions like food scarcity. *C. elegans*, with its rapid reproductive cycle and short generation time, faces frequent food shortages (Schulenburg and Felix, 2017). This nematode has developed adaptations to survive these events, with its response to food availability varying depending on the developmental stage when food becomes scarce [for review, see (Baugh and Hu, 2020; Rashid et al., 2020)]. Understanding these natural adaptive responses offers valuable insights into lifespan regulation.

**FIGURE 1:**
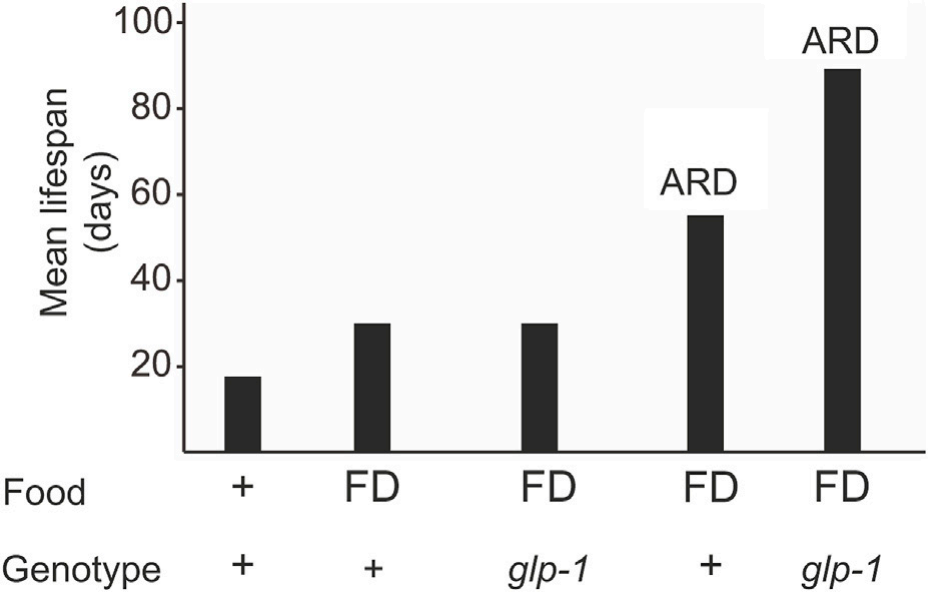
A *glp-1* mutation and ARD additively extend longevity. Animals can be subjected to diet restriction during adulthood or late larval stages (ARD). Food deprivation (FD) during adulthood does not increase lifespan in *glp-1* mutants but in ARD conditions.

Dietary restriction, which includes caloric restriction, intermittent fasting, and food deprivation, is a well-known condition that modulates lifespan (Loo et al., 2023). When food deprivation (FD) is limited to adulthood, it results in a 50% increase in lifespan (Figure 1) (Kaeberlein et al., 2006; Lee et al., 2006). Animals lacking proliferating germline (e.g., *glp-1* mutants) on FD do not show a further lifespan increase (Thondamal et al., 2014), indicating that the somatic gonad signal and the diet restriction pathways may converge to the same downstream mechanisms (Crawford et al., 2007; Thondamal et al., 2014).

*C. elegans* molts four times, going through larval stages named L1-L4 before becoming a reproducing adult. However, lack of food and other environmental conditions (e.g., pheromones, high temperatures) experienced by late L1 larvae results in the development of the L2d stage, followed by a non-feeding alternative stage called “dauer” (Golden and Riddle, 1984). In *C. elegans*, the dauer stage can last for up to a few months (Klass and Hirsh, 1976), a period during which the germline stops proliferating and remains undifferentiated. The process of dauer entry involves a rewiring of the metabolism (Penkov et al., 2020), including upregulation of genes involved in stress response and downregulation of genes involved in growth (Cohen et al., 2021). Despite active *glp-1* activity (Seidel and Kimble, 2015), germline stem cells arrest the cell cycle and require the PTEN tumor suppressor DAF-18 as well as LKB1/AMPK (AMP-activated protein kinase) signaling to maintain cell cycle quiescence (Ogg et al., 1998; Narbonne and Roy, 2006; Tenen and Greenwald, 2019). Larvae that hatch in the absence of food do not form dauers, but arrest development as L1 for up to 21 days (Johnson et al., 1984; Lee et al., 2012; Baugh, 2013). Germ cell arrest in this stage is also dependent on DAF-18 and AMPK (Fukuyama et al., 2006; Fukuyama et al., 2012), but does not require DAF-16 (Baugh and Sternberg, 2006; Fukuyama et al., 2006).

When starved in the late larval stages (e.g., L3 and L4), *C. elegans* reaches adulthood with a reduced number of germline cells that remain arrested in their cell division and differentiation (Angelo and Van Gilst, 2009; Schindler et al., 2014; Seidel and Kimble, 2015; Gerisch et al., 2020). This adult in reproductive diapause (ARD) lives almost three times the normal worm lifespan (Figure 1) (Angelo and Van Gilst, 2009; Gerisch et al., 2020). Once food becomes available, the germline starts to proliferate and the animal resumes to undergo a normal lifespan. *glp-1* mutants submitted to ARD live even longer (Figure 1), indicating that gonad signaling and ARD act through different pathways.

Molecular studies indicate some overlap between the germline pathway and ARD. Similar to *glp-1* mutants that lack a proliferating germline, ARD animals require HLH-30 and DAF-16 for lifespan extension (Gerisch et al., 2020). HLH-30 directly upregulates some genes involved in fat metabolism, such as *fat-5*, *fat-6*, *nhr-80*, and *lipl-3* (Gerisch et al., 2020). However, reduced activities of DAF-12, dafachronic acid, SKN-1, NHR-49, PHA-4, and HSF-1, which are necessary for the lifespan extension of *glp-1* mutants, had little or no effect on ARD lifespan (Gerisch et al., 2020). NHR-49, however, may be required for the initiation of ARD (Eustice et al., 2022).

In summary, food deprivation during late larval stages results in adults in reproductive diapause (ARD) that superficially resemble germline-ablated animals and mutants for germline proliferation. Although they share the lack of dividing germ cells, the extent of the longevity and molecular mechanisms seem to be different. It is possible that other ecologically relevant scenarios better mimic germline ablation. Nevertheless, it would be interesting to further investigate the molecular mechanisms underlying ARD to understand lifespan extension in a more natural context.

## The effects of germline removal in other nematodes

**FIGURE 2:**
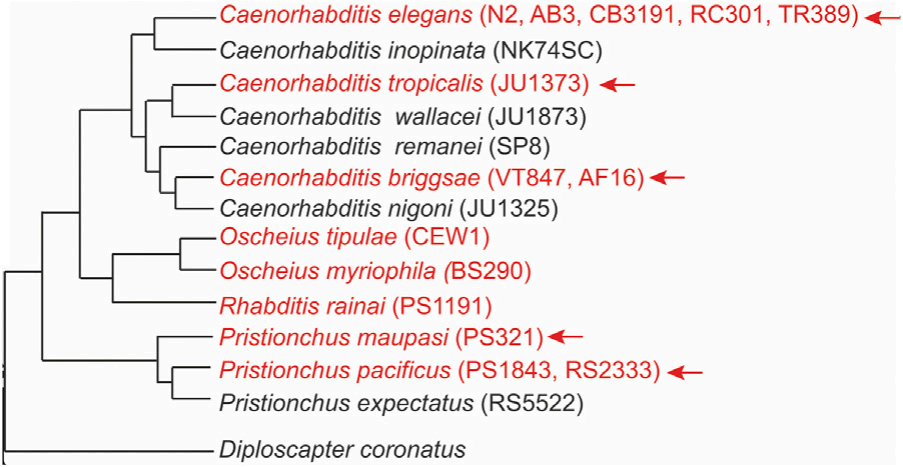
Germline ablation results in significant lifespan extension in hermaphrodites of most androdioecious species. Phylogeny of nematodes showing androdioecious species in red font. The arrow indicates the species that show significant lifespan extension after removing the germline, either by Z2/Z3 ablation or by performing *glp-1* RNAi. Strain names are in parenthesis. The phylogenetic tree was adapted from (Kiontke et al., 2005; Susoy et al., 2016; Stevens et al., 2019). *D. coronatus* is an outgroup and no germline ablation experiments were performed in this species.

To understand the generality of mechanisms behind lifespan extension in mutants lacking germline, a comparative analysis is necessary. Recent research has shown that early reproductive efforts are linked to pathologies emerging at post-reproductive age. Hermaphrodites from species of the *Caenorhabditis* and *Pristionchus* genera that can reproduce with males (androdioecious species) die sooner than their relatives that have females and males (gonochoristic species) (Kern et al., 2023) (Figure 2). This earlier death of hermaphrodites is largely attributed to the significant amount of energy expended in producing yolk (Kern et al., 2023).

In most androdioecious species studied, removing the germline in hermaphrodites led to a significant increase in lifespan (Hsin and Kenyon, 1999; Patel et al., 2002; Kern et al., 2023). In contrast, corresponding experiments in gonochoristic sibling species resulted in little or no lifespan extension in females (Hsin and Kenyon, 1999; Patel et al., 2002; Kern et al., 2023). The germline removal in hermaphrodites may suppress “reproductive death,” a rapid death process typically caused by the intense demands of reproduction (Gems et al., 2021). This type of death is considered programmatic rather than random, as signals from the somatic gonad can modulate it. Indeed, removing the entire gonad in hermaphroditic species does not delay senescence onset, whereas germline ablation does, indicating counteracting signals between the gonad tissues (Kern et al., 2021).

In hermaphrodites of androdioecious species, a common senescent pathology during aging is excessive yolk production by the intestine (Ezcurra et al., 2018; Sornda et al., 2019; Kern et al., 2023). This yolk overproduction leads to intestinal atrophy, driven by extensive autophagy and lipophagy, which are essential processes for generating the biomass necessary for lipoprotein synthesis (Ezcurra et al., 2018). In contrast, virgin females of gonochoristic species do not exhibit intestinal atrophy or yolk accumulation in the pseudocoelom, typically resulting in a longer lifespan compared to their androdioecious sibling species (Kern et al., 2021). However, upon mating, these females exhibit aging patterns and pathologies similar to those of hermaphrodites. It has been proposed that the abundant production of yolk may be adaptive in hermaphrodites, as lipoproteins can be released into the environment to serve as a nutritional source for the offspring (Kern et al., 2021). Mated females release only minimal amounts of yolk, and mating in hermaphrodites similarly decreases the levels of yolk they vent. This reduction is hypothesized to result from the absorption of yolk into oocytes that are fertilized later (Kern et al., 2023).

Germline removal in non-*Caenorhabditis* nematodes, such as *P. pacificus*, results in gene expression changes and phenotypes that are similar to those found in *C. elegans*. These include the accumulation of fat and upregulation of genes involved in fat metabolism (e.g., *fat-7*), enrichment for DAF-16 targets, and downregulation of the insulin pathway (Rae et al., 2012). However, whether those changes are functionally relevant for influencing lifespan in *P. pacificus* is unclear. It would be interesting to further investigate the *Oscheius* species since ablation of the germline cells in two of the hermaphroditic species does not seem to extend lifespan (Patel et al., 2002) (Figure 2). It is possible these species recently evolved hermaphroditism and have yet to develop mechanisms associated with reproductive death. Nematode species that have both hermaphrodites and females may also provide valuable insights (Chaudhuri et al., 2011; Kanzaki et al., 2017).

## Reproduction and the evolution of aging

The concept that there is a trade-off between the probability of death and reproduction underpins the evolutionary theory of aging (Maklakov and Immler, 2016). According to the “disposable soma” theory, there is a competition for resources between somatic maintenance and reproduction (Kirkwood, 1991; Lemaitre et al., 2015). However, removing germ cells not only extends lifespan but also enhances resistance to a wide range of environmental and biological stressors (Hsin and Kenyon, 1999), contradicting this theory. Lifespan extension as a result of germ cell removal is not restricted to nematodes. The fruitfly *Drosophila* without proliferating germline stem cells shows increased longevity (Flatt et al., 2008), and gonad removal in vertebrates such as fish also results in extended lifespan (Gems et al., 2021). Likewise, human eunuchs have been reported to live about 15 years longer than non-castrated men (Min et al., 2012), although the accuracy of these historical records has been challenged on methodological grounds (Le Bourg, 2015).

Critical to understanding aging is to identify the proximal causes. The disposable soma theory assumes that resources are required to repair somatic tissues and that the accumulation of damage is the proximate cause of aging. Nevertheless, the concept that aging is driven by molecular damage from oxidative damage (Gems and Doonan, 2009; Perez et al., 2009; Van Raamsdonk and Hekimi, 2010), or change in telomere length (Raices et al., 2005; Cook et al., 2016) [but see (Joeng et al., 2004)] lacks empirical support, at least in nematodes. In fact, it has been suggested that many of the elements identified as “hallmarks of aging” (López-Otín et al., 2013; Lopez-Otin et al., 2023), which include cellular damage, cannot be generalized to many organisms including *C. elegans* (Gems and de Magalhães, 2021). It would be useful to identify what are the primary, secondary, and tertiary causes of aging, as well as how they give rise to aging (Gems and de Magalhães, 2021).

From the work on comparison between the rate of aging in nematodes with different modes of reproduction (Kern et al., 2023), hermaphrodites seem to undergo a mechanistically non-stochastic (programmatic) aging process (Blagosklonny, 2006). This is an alternative theory of aging, which proposes that exaggerated investment in reproduction leads to post-reproductive senescent pathologies (Kern and Gems, 2022). When this investment is prevented by germline removal, hermaphrodites live as long as the females of sister species (which do not have programmatic aging) (Kern et al., 2023). Interestingly, the most significant lifespan increases following germline removal have been observed in semelparous animals, which show a terminal reproductive effort that leads to their death (Kern and Gems, 2022). It is thus likely that interventions proven to significantly extend the lifespan of *C. elegans*, such as mutations stopping germline proliferation or the removal of germline cells, might be specific to organisms that experience reproductive death.

Although not addressing directly the evolution of aging, a potentially interesting avenue of research would be to compare the pattern and mechanisms of aging between closely related species. For instance, it would be interesting to determine if transcription factors known to be active in germline-less hermaphrodites are also active in females of sister species, and whether interventions found to increase the lifespan in wild-type hermaphrodites (e.g., constitutive expression of *lipl-4*) has the same effect on females. In species with no reproductive death such as *Drosophila*, similar changes to *C. elegans* occur after germline ablation, such as lifespan extension, fat storage, and lipid enzyme regulation (Steinbaugh et al., 2015; Rodrigues et al., 2023). These results would suggest the conservation of mechanisms of lifespan extension, but more research is required to determine this.

## Concluding remarks and outlook

Some mechanisms explaining the increased longevity of germline-less *C. elegans* are seemingly contradictory. For example, while high autophagy is thought to shorten lifespan in wild-type animals by leading to the consumption of their gut (Ezcurra et al., 2018; Kern et al., 2023), long-lived germline-less individuals also exhibit high levels of autophagy (Lapierre et al., 2011). This discrepancy may be due to different uses of autophagy products in these scenarios, resulting in different outcomes. For instance, *glp-1* animals produce more yolk protein than wild-type animals on the first day of adulthood (Steinbaugh et al., 2015). However, yolk levels increase substantially with age in wild-type worms, peaking around the seventh day of adulthood (Ezcurra et al., 2018). This suggests that the elevated yolk in germline-less animals might not result from the same harmful autophagic gut-to-yolk biomass conversion seen in wild-type animals but from the synthesis of fat from other sources. Indeed, a *glp-4* mutation abrogates intestinal atrophy (Ezcurra et al., 2018), suggesting this autophagic process may not function in the same manner in germline-less animals. An additional possibility is that other mechanisms triggered by the absence of a proliferating germline could compensate for the harmful effects of high autophagy seen in animals with an intact germline.

Most studies have primarily focused on possible pro-longevity factors mediated by the somatic gonad. However, there is now an increasing interest in exploring pro-aging signals mediated by the germline. The Hedgehog signaling pathway, a conserved regulator of animal development (Ingham et al., 2011), has been recently implicated in this process (Shi and Murphy, 2023). Germline hyperactivity, triggered by mating, activates the Hedgehog pathway and also mediates the lifespan in other invertebrates (Rallis et al., 2020).

For a comprehensive understanding of how aging mechanisms work in *C. elegans,* further research should systematically involve both sexes (Ancell and Pires-daSilva, 2017). For some longevity treatments, there are clear differences between the sexes (Honjoh et al., 2017). Diet restriction, for example, extends the lifespan of *C. elegans* hermaphrodites, but not of males. The response to diet restriction is mediated by the terminal effector of sex determination TRA-1 (Honjoh et al., 2017), a transcription factor that promotes longevity in hermaphrodites by upregulating some isoforms of *daf-16* (Hotzi et al., 2018). The lower. TRA-1 activity in males leads to higher expression of the nuclear receptor DAF-12, resulting in a weaker DR response (Honjoh et al., 2017).

There is still a large gap in our understanding of the relationship between molecular changes, lifespan, and causes of death. Some of the remaining broader questions include the proximate causes of aging and the causes of pathologies of aging that result in death (Gems and de Magalhães, 2021), and commonalities of mechanisms of lifespan extension under different conditions (e.g., dauer, L1 arrest, ARD, germline-less) within and between species. For example, it is unclear if regulators of germ cell quiescence in L1 and dauer (DAF-18/PTEN and AMPK) also have a role in the lifespan extension of germline ablated or germ-cell nematodes. It is also unclear why many of the genes necessary for lifespan extension in germline-less animals (e.g., DA/DAF-12 signaling, TCER-1, and lipid metabolism genes) are also required for reduced fecundity in post-dauer adults (Adams et al., 2022). The identity of the signals that mediate the communication between the soma and germline cells (Conine and Rando, 2022; Ow and Hall, 2024), and that could influence lifespan (Hsin and Kenyon, 1999), are also largely unexplored. Answering these questions will guide the research to formulate hypotheses that could be tested experimentally. For instance, while the accumulation of cellular damage has been a popular paradigm for explaining the primary cause of aging, experimental evidence has raised doubts about its validity (de Magalhaes and Church, 2006; Gems and Doonan, 2009; Perez et al., 2009). Thus, new theories that consider biological constraints (Gems and Kern, 2022) and that can unite proximal with the ultimate causes of aging (Gems, 2022) are welcome.
